# Treadmill exercise within lower-body negative pressure attenuates simulated spaceflight-induced reductions of balance abilities in men but not women

**DOI:** 10.1038/npjmgrav.2016.22

**Published:** 2016-06-30

**Authors:** Timothy R Macaulay, Brandon R Macias, Stuart MC Lee, Wanda L Boda, Donald E Watenpaugh, Alan R Hargens

**Affiliations:** 1 Department of Orthopaedic Surgery, University of California, San Diego, San Diego, CA, USA; 2 Cardiovascular Laboratory, Wyle Science, Technology and Engineering Group, Houston, TX, USA; 3 Department of Kinesiology, Sonoma State University, Rohnert Park, CA, USA; 4 Department of Integrated Physiology, University of North Texas, Fort Worth, TX, USA

## Abstract

Spaceflight causes sensorimotor adaptations that result in balance deficiencies on return to a gravitational environment. Treadmill exercise within lower-body negative pressure (LBNP) helps protect physiological function during microgravity as simulated by bed rest. Therefore, we hypothesized that treadmill exercise within LBNP would prevent balance losses in both male and female identical twins during 30 days of 6° head-down tilt bed rest. Fifteen (seven female and eight male) identical twin sets participated in this simulation of microgravity. Within each twin pair, one twin was randomly assigned to an exercise group that performed 40 min of supine treadmill exercise within LBNP set to generate 1.0–1.2 body weight, followed by 5 min of static feet-supported LBNP, 6 days per week. Their identical sibling was assigned to a non-exercise control group with all other bed rest conditions equivalent. Before and immediately after bed rest, subjects completed standing and walking rail balance tests with eyes open and eyes closed. In control subjects, standing rail balance times (men: −42%, women: −40%), rail walk distances (men: −44%, women: −32%) and rail walk times (men: −34%, women: −31%) significantly decreased after bed rest. Compared with controls, treadmill exercise within LBNP significantly attenuated losses of standing rail balance time by 63% in men, but the 41% attenuation in women was not significant. Treadmill exercise within LBNP did not affect rail walk abilities in men or women. Treadmill exercise within LBNP during simulated spaceflight attenuates loss of balance control in men but not in women.

## Introduction

Spaceflight exposure compromises balance. For example, during Skylab missions (28–84 days), exposure to microgravity reduced astronauts’ abilities to balance with both feet on a narrow rail by up to 90%.^1^ More recent evaluations of balance using dynamic posturography tests capable of isolating visual, vestibular, and somatosensory inputs confirm that spaceflight reduces postural stability and standing balance control.^2,3^

Because astronauts do not operate in a static environment, walking balance tests provide additional important data, integrating the visual, vestibular, somatosensory, and musculoskeletal systems. For example, walking balance tests at the NASA (National Aeronautics and Space Administration) Johnson Space Center involve obstacle courses that require activation of functional balance and postural stability. Astronauts demonstrate 48% slower obstacle course times on return from spaceflight,^4,5^ as well as modified head-to-trunk movement strategies, especially when turning a corner.^6^ Since scores on dynamic posturography tests and functional mobility tests provide independent balance data,^5^ utilizing both standing and walking balance tests may allow better detection of balance deficiencies than using either test alone.

Sensorimotor adaptations to spaceflight conditions are inappropriate for gravitational environments. Whether in Earth’s or Mars’ gravitational environment, astronauts must maintain upright postural stability to perform operational tasks and prevent falls, especially during an emergency vehicle egress. Utilization of spaceflight countermeasures that protect balance could reduce the fall and fracture risk created by long-duration spaceflight.

Exercise is commonly used as a countermeasure to prevent physiological deconditioning during spaceflight. Exercise protocols on the International Space Station (ISS) are effective in preventing some of the physiological deconditioning that occurs in microgravity. For example, periodic resistive exercises on the ISS target weight-bearing bones and help maintain some bone mineral density and lean body mass during 4–6 months of spaceflight.^7^ However, current exercise countermeasures on the ISS during long-duration spaceflight do not prevent decrements in standing and walking balance abilities.^5^

Bed rest with 6° of head-down tilt (HDT) is an accepted model for ground-based simulations of spaceflight to study physiological deconditioning and countermeasure efficacy.^8^ Even though bed rest subjects continue to experience Earth’s gravity in transverse axes (Gx and Gy), they do not receive the usual upright, daily axial (Gz) body loading and proprioceptive stimuli. HDT bed rest has proven useful in distinguishing spaceflight-induced sensorimotor adaptations from the adaptive responses that occur in response to deconditioning alone.^6,8,9^ For example, deconditioning alone, as a result of physical inactivity and musculoskeletal unloading on Earth, reduces musculoskeletal strength and adversely affects postural muscle function in the legs by altering proprioceptive feedback and motor control.^10^ In comparison, microgravity exposure causes greater systemic musculoskeletal unloading, as well as cardiovascular adaptations from loss of normal hydrostatic pressures^11^ and vestibulo-spinal adaptations from reduced otolith stimulation.^12^ HDT bed rest simulates to a large extent the redistribution of body fluids, vascular volume loss, bone demineralization and musculoskeletal unloading that are experienced during actual microgravity.^13^

Short-duration exposure to gravity or simulated gravity during HDT bed rest protects some measures of postural stability and balance control. For example, in a 5-day HDT bed rest study with male participants, standing for 25 min per day increased postural stability and produced fewer falls after bed rest than in non-weight-bearing bed rest controls.^9^ Therefore, short-duration exposures to axial (Gz) loading and normal standing hydrostatic pressure gradients during simulated spaceflight may protect balance control. Similar loading configurations can be provided by lower-body negative pressure (LBNP). Two modes of LBNP have been used in space, both of which provide an orthostatic stress for assessing cardiovascular responses and protecting against post-flight orthostatic intolerance.^14^ The first use of LBNP employs a saddle such that the user’s legs are unloaded and independent, whereas the second use of LBNP, feet-supported LBNP, compresses the user’s feet against the bottom of the LBNP device with a force proportional to the level of negative pressure, thus loading the musculoskeletal system, as well as the cardiovascular system. In one study, daily supine feet-supported LBNP (1–2 h per day at −28 mm Hg) protected static (anterior–posterior and lateral balance) and some dynamic standing balance stability in men during 30 days of HDT bed rest.^15^ Because LBNP can be used as a form of artificial gravity, treadmill exercise within LBNP is a potential countermeasure for simulating upright exercise on Earth. This third form of LBNP, which has not been used in space, provides gravity-like loads to the musculoskeletal and cardiovascular systems during HDT bed rest.^16^ Our previous reports document that treadmill exercise within LBNP, followed by brief static, feet-supported LBNP protects against deconditioning in various physiological systems during HDT bed rest, including upright exercise capacity,^17,18^ orthostatic tolerance,^19^ spine function,^20^ bone homeostasis,^21,22^ and muscular strength.^23^ Furthermore, we have previously demonstrated that this LBNP exercise protocol maintains sprint performance in both men and women during HDT bed rest,^17,18^ suggesting that this exercise countermeasure may help protect functional dynamic balance. It is unclear, however, whether supine treadmill exercise within LBNP protects standing and walking balance abilities during 30 days of HDT bed rest.

The purpose of this study was to determine the efficacy of treadmill exercise within LBNP as a countermeasure for balance losses experienced during microgravity simulated by 6° HDT bed rest. We hypothesized that treadmill exercise within LBNP followed by static feet-supported LBNP would prevent losses in balance abilities in both female and male identical twins during 30 days of 6° HDT bed rest. This study is one of a series to investigate the use of treadmill exercise within LBNP as a countermeasure to spaceflight-induced deconditioning.

## Results

Both men and women (exercise and control groups) had significantly longer single-leg rail balance times with eyes open than with eyes closed (*P*<0.01), but there was no difference between eyes open and eyes closed performance decrements after 30 days of HDT bed rest. In addition, there was no difference between balance times on the left or right legs (*P*>0.05). For these reasons, and to test our original hypothesis, single-leg rail balance data are presented as an average of the eyes open, eyes closed, left leg, and right leg tests. Similarly rail walk distance and time were significantly better with eyes open than with eyes closed (*P*<0.01), but there was no difference between eyes open and eyes closed performance decrements after HDT bed rest. Therefore all rail walk data are presented as an average of the eyes open and eyes closed tests. The summary statistics that accompany the following results are presented in Table 1.

### Single-leg rail balance

**Figure 1:**
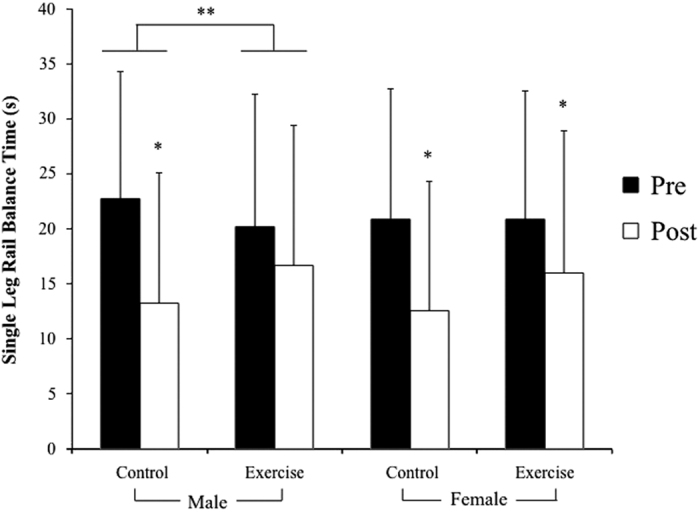
Pre- and post-bed rest single-leg rail balance times (mean±s.d.) for each group. Data are pooled from four test conditions: eyes open, eyes closed, left leg, and right leg. The male control group, female control group, and female exercise group had significantly shorter single-leg rail balance times after bed rest than before (*P<0.01). The decrease in balance time from pre- to post-bed rest was significantly smaller in the male exercise group than in the male control group (**P=0.037).

**Figure 2:**
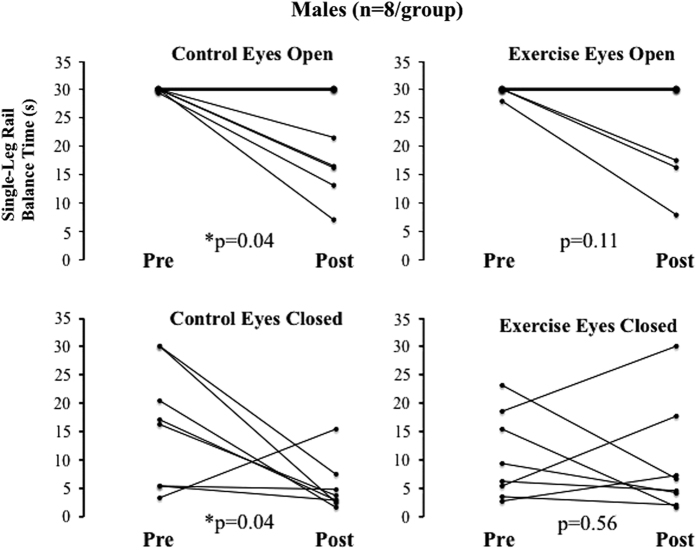
Individual changes from pre- to post-HDT bed rest in single-leg rail balance time for each male group, with eyes open and with eyes closed. Each graph represents data from eight male subjects. Right leg and left leg balance times were averaged. *Group balance time significantly decreased after bed rest (*P*<0.05). HDT, head-down tilt.

Treadmill exercise within LBNP attenuated losses in single-leg rail balance abilities in men after 30 days of HDT bed rest. Before bed rest there was no difference between the male control and exercise groups’ single-leg rail balance time. After bed rest, single-leg rail balance time was significantly shorter, relative to values before bed rest, in the male control group (*P*=0.001) but not in the male exercise group (Figure 1). Comparing balance losses from pre- to post-bed rest, balance time decreased significantly less in the exercise group than in the control group (*P*=0.037) (Figure 1). In addition, the percentage of subjects×tests that reached the 30-s maximum decreased less (from pre- to post-bed rest) in the male exercise group (56–41%) than in the male control group (from 66 to 28%). Single-leg rail balance times were further analyzed because of the significant effects of eye conditions and treadmill exercise within LBNP, and to evaluate balance time changes in individual subjects. With left and right leg balance times averaged, the number of combined tests (8 eyes open and 8 eyes closed) that reached the 30-s maximum decreased less (from pre- to post-bed rest) in the male exercise group (7/16 to 6/16) than in the male control group (10/16 to 3/16). Furthermore, control group balance time significantly decreased in both the eyes open (*P*=0.04) and eyes closed (*P*=0.04) conditions, whereas exercise group balance time did not significantly change (eyes open: *P*=0.11, eyes closed: *P*=0.56) (Figure 2). Therefore, treadmill exercise within LBNP improved the proportion of balance test completion and prevented, on average, 63% ((ΔControl group−ΔExercise group)/(ΔControl group)) of the losses in male single-leg rail balance time during HDT bed rest.

**Figure 3:**
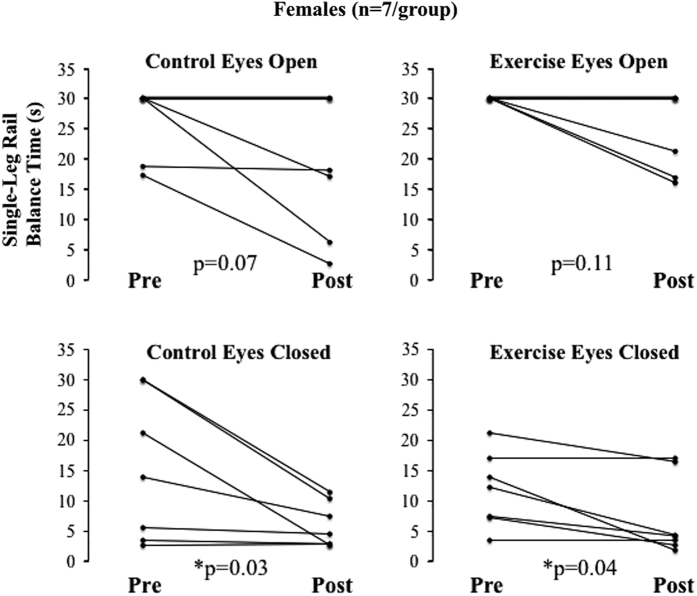
Individual changes from pre- to post-HDT bed rest in single-leg rail balance time for each female group, with eyes open and with eyes closed. Each graph represents data from seven female subjects. Right leg and left leg balance times were averaged. *Group balance time significantly decreased after bed rest (*P*<0.05). HDT, head-down tilt.

Single-leg rail balance abilities in women, however, were not protected by treadmill exercise within LBNP during 30 days of HDT bed rest. Before bed rest there was no difference between the female control and exercise groups’ single-leg rail balance time. After bed rest, single-leg rail balance time was significantly shorter, relative to values before bed rest, in both the female control group (*P*=0.004) and the female exercise group (*P*=0.005) (Figure 1). The pre- to post-bed rest decreases in balance time between the female control and exercise groups were not significantly different (Figure 1). Therefore, despite the fact that there was a 41% difference between single-leg rail balance losses in the exercise group than in the control group ((ΔControl group – ΔExercise group)/(ΔControl group)), treadmill exercise within LBNP had no significant protective effect. Similar to men, the percentage of female subjects×tests that reached the 30-s maximum decreased less (from pre- to post-bed rest) in the female exercise group (57–43%) than in the female control group (57–29%). To permit comparisons to men, female single-leg rail balance times were also further analyzed (Figure 3). With left and right leg balance times averaged, the number of combined tests (7 eyes open and 7 eyes closed) that reached the 30-s maximum decreased less (from pre- to post-bed rest) in the female exercise group (7/14 to 4/14) than in the female control group (7/14 to 3/14). Furthermore, both the control and exercise groups’ balance times significantly decreased in the eyes closed conditions (*P*=0.03 and *P*=0.04, respectively) but not in the eyes open condition (*P*=0.07 and *P*=0.11, respectively) (Figure 3).

### Rail walk

**Figure 4:**
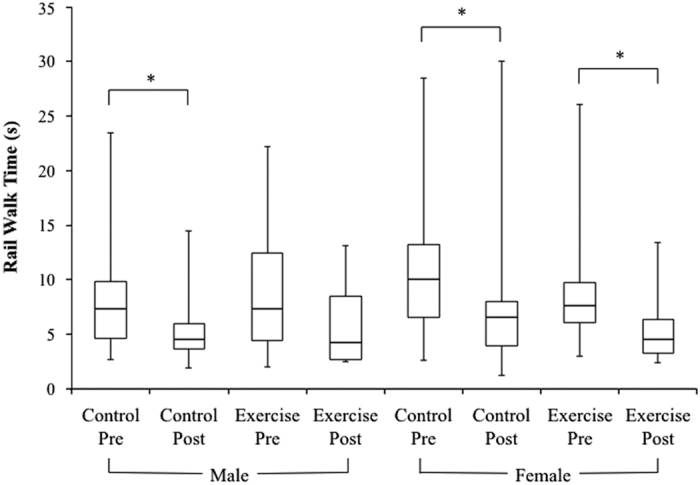
Box-and-whisker plots of pre- and post-bed rest rail walk times (data pooled from eyes open and eyes closed test conditions) for each group. The bottom whisker indicates the minimum value in the data set, the bottom of the box indicates the first quartile, the horizontal line within the box indicates the median, the top of the box indicates the third quartile and the top whisker indicates the maximum. The male control group, female control group, and female exercise group had significantly shorter rail walk times after bed rest than before (*P<0.05). The pre- to post-bed rest decreases in balance time were not significantly different between the male control and exercise groups and between the female control and exercise groups.

**Figure 5:**
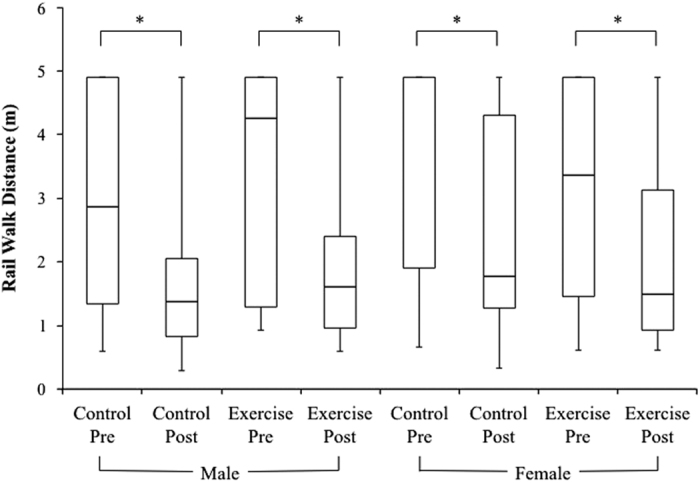
Box-and-whisker plots of pre- and post-bed rest rail walk distances (data pooled from eyes open and eyes closed test conditions) for each group. The bottom whisker indicates the minimum value in the data set, the bottom of the box indicates the first quartile, the horizontal line within the box indicates the median, the top of the box indicates the third quartile, and the top whisker indicates the maximum. All groups had significantly shorter rail walk distances after bed rest than before (*P<0.04). Exercise did not improve rail walk distance in both male and female subjects after bed rest, as compared with controls.

Treadmill exercise within LBNP did not have a significant effect on rail walk abilities in men or women after 30 day of HDT bed rest. In men, treadmill exercise within LBNP did not protect rail walk time. Before bed rest, there was no difference between the male control and exercise groups’ rail walk time. After bed rest, rail walk time was significantly shorter, relative to values before bed rest, in the male control group (*P*=0.023) but not in the male exercise group (Figure 4). However, the pre- to post-bed rest decreases in rail walk time between the male control group and male exercise group were not significantly different (Figure 4). Treadmill exercise within LBNP also did not have an effect on rail walk distance in men. Before bed rest, there was no difference between the male control and exercise groups’ rail walk distance. After bed rest, rail walk distance was significantly shorter, relative to values before bed rest, in both the male control group (*P*=0.012) and the male exercise group (*P*=0.016) (Figure 5). The pre- to post-bed rest decreases in rail walk distance between the male control group and male exercise group were not significantly different (Figure 5).

In women, treadmill exercise within LBNP did not have an effect on rail walk time. Before bed rest, there was no difference between the female control and exercise groups’ rail walk time. After bed rest, rail walk time was significantly shorter, relative to before bed rest, in both the female control group (*P*=0.048) and the female exercise group (*P*=0.009) (Figure 4). The pre- to post-bed rest decreases in rail walk time between the female control group and female exercise group were not significantly different (Figure 4). In addition, treadmill exercise within LBNP did not have an effect on rail walk distance in women. Before bed rest, there was no difference between the female control and exercise groups’ rail walk distance. After bed rest, rail walk distance was significantly shorter, relative to before bed rest, in both the female control group (*P*=0.004) and the female exercise group (*P*=0.036) (Figure 5). The pre- to post-bed rest decreases in rail walk distance between the female control group and female exercise group were not significantly different (Figure 5).

## Discussion

Our study’s principal finding is that treadmill exercise within LBNP protected single-leg rail balance time by 63% in men during 30 days of HDT bed rest, relative to identical twin controls. Treadmill exercise within LBNP did not, however, provide significant protection for female single-leg rail balance abilities, male rail walk abilities, or female rail walk abilities. These data support our hypothesis that treadmill exercise within LBNP protects the standing balance abilities of men. However, our data fail to support our hypothesis that treadmill exercise within LBNP protects the walking balance abilities of men and the standing and walking balance abilities of women. The protection of balance abilities in men provides additional evidence that treadmill exercise within LBNP helps mitigate some of the physiological deconditioning that occurs during simulated microgravity.^17,19,20^ The observed impairments in standing and walking balance abilities in this study are consistent with other bed rest^8,9,15,24^ and spaceflight^1–5,12,25^ investigations.

We observed in control subjects that 30 days of HDT bed rest decreased male and female single-leg rail balance times by 42% and 40%, respectively. Previous studies have demonstrated similar decrements in standing balance control after HDT bed rest: 31% decrease in men after 30 days^15^ and 46% decrease in women after 60 days.^24^ In addition, these balance decrements are similar to, but less than, those observed after spaceflight. For example, 28–84 days of spaceflight reduced standing rail balance abilities by 40–90%.^1^ HDT bed rest may affect balance to a lesser degree than spaceflight because a gravitational vector still exists during bed rest. Further discrepancies between spaceflight and bed rest balance decrements may be due to the type of rail balance test performed and the duration of unloading. For example, the astronauts in the aforementioned study^1^ balanced on both feet in a tandem heel-to-toe arrangement on rails of varying width up to 5.72 cm. In contrast, subjects in our study balanced with one foot on a wider (7.6 cm) rail. Although balancing on one foot reduces the base of support and forces subjects to shift their center of gravity, standing on a wider rail and having a leg free to counterbalance may aid balance. In addition, the astronauts in the aforementioned study^1^ were in space up to 84 days, compared with 30 days of HDT bed rest for our identical twins.

To our knowledge, this is the first study to document the role of sex in standing and walking balance abilities after simulated spaceflight. Treadmill exercise within LBNP prevented 63% of standing balance losses in men during 30 days of HDT bed rest. Similarly, feet-supported LBNP alone (1–2 h per day at −28 mm Hg) protected standing balance (during anterior–posterior and lateral sway) in men by 52% during 30 days of HDT bed rest.^15^ However, treadmill exercise within LBNP did not significantly protect standing balance abilities in women. This conclusion is consistent with the findings of a previous study, which demonstrated that a combined exercise countermeasure involving treadmill exercise within LBNP, static feet-supported LBNP, and HDT flywheel resistance training did not prevent balance losses in women during 60 days of HDT bed rest.^24^ Even though the female exercise subjects in our present study were in HDT for half the duration of the 60-day bed rest study and were exposed to 2 more hours of treadmill exercise within LBNP per week, they still experienced an average 23% loss in standing balance control. More sensitive and more involved tests such as dynamic posturography may better distinguish the ways in which bed rest, and treadmill exercise within LBNP during bed rest, affect standing balance because these types of tests allow independent assessment of sensory contributions to balance.^26^

HDT bed rest impairs rail walk performance in both men and women. In addition, subjects appear more cautious during rail walk tests after bed rest than before bed rest. Other tests of walking balance have similarly reported a 27% decrease in functional walking balance abilities (for subjects who did not have sore feet) measured on an unstable obstacle course after 42–90 days of HDT bed rest.^8^ In comparison, astronauts’ performance in this obstacle course decreases by 48% after 185 days of spaceflight,^4^ even though astronauts exercise in microgravity. Treadmill exercise within LBNP in this study does not fully protect all variables of rail walk abilities in men and women during 30 days of HDT bed rest. Rail walk time is significantly shorter in the male control group after bed rest (as compared with before bed rest) and not significantly shorter in the male exercise group after bed rest. However, calculated pre- to post-bed rest decreases in male rail walk times were not significantly different between the control and exercise groups. Therefore our results do not provide sufficient evidence that treadmill exercise within LBNP protects rail walk time in men. The lack of significance of these results may be due to large variability in the data, as demonstrated by the large s.d. Similarly in a different HDT bed rest study, alternative supine exercise countermeasures (involving both resistance and aerobic exercise) did not prevent decreases in the time needed to complete an unstable obstacle course after bed rest.^27^ That study did, however, use the obstacle course performance of non-bed rest subjects to conduct a trend analysis, demonstrating that exercise partially mitigates the effects of HDT bed rest. In addition, they found that their exercise countermeasure improved the rate of post-bed rest balance recovery. More sensitive walking tests, comparisons to non-bed rest subjects, and measurements of balance recovery may help us better distinguish the effects of treadmill exercise within LBNP during HDT bed rest.

In addition to the well-characterized alterations in somatosensory, vestibular, and visual sensory systems that occur after bed rest and microgravity exposures,^2,3,8,9^ balance abilities may also be affected by presyncopal symptoms caused by orthostatic intolerance. Orthostatic intolerance is present after both real and simulated microgravity exposures, and is associated with hypovolemia, cardiac remodeling, and decreased systolic and mean arterial blood pressure.^19,28,29^ Dizziness due to hypotension can simulate self-motion, creating a visual sensory discordance that increases variability in locomotion coordination patterns.^30^ Our previous studies document that the treadmill exercise within LBNP countermeasure is effective in attenuating orthostatic intolerance and cardiac atrophy during HDT bed rest.^19,29^ Improving orthostatic intolerance likely helps increase balance performance due to the absence of dizziness and tunnel vision.

Female standing and walking balance abilities are not protected by treadmill exercise within LBNP during 30 days of HDT bed rest. It is well-known that women tend to have less knee flexion and hip abduction and external rotation strength relative to body size in comparison to men.^31^ Therefore, one may postulate that muscle losses after bed rest affect female balance control more than male balance control. However, preliminary results from our leg strength tests in this study suggest that women experience smaller decreases in isokinetic knee strength than men after bed rest.^32^ Furthermore, because these data also suggest that treadmill exercise within LBNP preserves leg lean muscle mass and strength in both men and women, balance losses are likely not caused by muscle atrophy or strength losses.

Women experience greater orthostatic intolerance than men post-bed rest, which may contribute to balance impairments. Orthostatic intolerance is generally greater in women after spaceflight and bed rest because of larger plasma volume losses, greater vascular compliance, and insufficient adrenergic responses.^28,33^ In a separate bed rest study, exercise countermeasures involving treadmill exercise within LBNP and flywheel resistive exercise did not completely protect against orthostatic intolerance or balance loss in women during 60 days of HDT bed rest.^24,34^ In the current study, orthostatic tolerance was measured before and after bed rest prior to balance testing. Unfortunately, our previously-reported orthostatic intolerance data for these identical twin subjects does not decipher the role of sex in orthostatic intolerance.^19^ Female identical twins in our study did indeed report more symptoms than the male identical twins in the study on initial standing after bed rest. However, the extent to which these symptoms of orthostatic intolerance affect balance is unclear.

Multidisciplinary and integrated countermeasures are important because sensorimotor processes have widespread interactions with other physiological and psychological processes.^35^ Treadmill exercise within LBNP is an integrated countermeasure to counteract musculoskeletal loss, cardiovascular deconditioning, reduced ambulation ability, and the adverse effects of head-ward fluid shifts in microgravity.^17–20,22^ To our knowledge, there are currently no published data on the effectiveness of in-flight countermeasures in combating balance losses because all astronauts perform exercise countermeasures in space. Currently crew members utilize a bungee cord-treadmill exercise device on the ISS. Microgravity necessitates that several latex rubber cords attached to a shoulder and waist harness hold the user to the treadmill surface and provide partial musculoskeletal loads. Loading, however, is restricted to about 70% of body weight by the user’s tolerance for the harness’s compression forces on the shoulders and waist. Therefore, according to data from Expeditions 6–12 (2002–2006), walking and running on the spaceflight treadmill in microgravity with maximum tolerated loading produce ~77% and 75% less, respectively, of in-shoe forces as compared with walking and running on Earth.^36^ Because the reduction of lower limb musculoskeletal forces and tissue stresses is equivalent to the decrease in ground reaction forces,^37,38^ these stimuli may not be sufficient for maintaining lower limb strength and balance control during prolonged spaceflight. Although recent advances in harness design have made treadmill exercise with high gravity replacement loads more comfortable,^39^ external forces during exercise are still on the order of 52–80% of body weight,^40^ with bungee stiffness being one of the major limiting factors due to its effects on running mechanics.^41^ Regardless, running on the current spaceflight treadmill does not provide gravity-like hydrostatic pressure gradients that are important for maintaining Earth-like hemodynamics.^11^

However, treadmill exercise within LBNP in the present study provides subjects with comfortable lower-body forces of 1.0–1.2 body weight at rest, even higher body weight loads with exercise, as well as comfortable spinal loads using shoulder straps connected to the LBNP waist seal. Thus, treadmill exercise within LBNP produces ground reaction forces, intramuscular contraction pressures, spinal loads, metabolic responses, and axial tibial forces similar to those produced by normal upright locomotion on Earth.^16,38,42^ This is important because locomotion is a critical aspect of training to improve walking balance abilities in a novel gravity environment.^43^ Therefore the production of tissue stresses and neuromuscular activation patterns similar to those of upright walking and running on Earth should help prevent losses of balance abilities during actual microgravity.

A future direction to improve balance countermeasures involving treadmill exercise within LBNP is to incorporate sensory variation to facilitate adaptive generalization. This technique for improving sensorimotor function is based on the idea that exposure to sensory variation during previous training for a specific motor task allows humans to better adapt learned motor skills to a novel sensorimotor environment.^43^ Adding adaptive challenges such as virtual reality, bilateral treadmill speed changes, and alternating support surfaces to the treadmill exercise within LBNP countermeasure may improve exercise subjects’ ability to readapt to locomotion in Earth’s gravity.

The beneficial effects of treadmill exercise within LBNP on standing balance in men may be less substantial than on other previously demonstrated physiological parameters. However, our statistical analyses indicate that this balance protection is indeed significant. Although the sample size in this study may have been a limitation, we addressed this potential issue by using identical twins as a way of increasing statistical power.^44^ Finally, although the mean effects of treadmill exercise within LBNP on balance are small, the individual consequences may be critical, especially when projected to long-duration spaceflight missions.

One limitation of the present study involves the time of the test. Our balance tests follow an orthostatic tolerance test and sprint test. Although subjects are kept supine between tests, some re-adaptation to upright posture may have occurred before the balance tests were performed. Learning effects may have been present during balance testing, but they were likely too small to affect the results, especially because only Trial 1 values were used. During single-leg rail balance tests, some subjects who performed a second trial did have longer balance times in that trial compared with the first (in the same leg and eye conditions), but not to a significant level (*P*>0.05). Furthermore, because there was no difference between balance times on the left and right legs (*P*>0.05), and left leg tests always preceded the right, any small learning effects likely did not carry over between legs or between different tests. It is unclear whether single-leg rail balance learning affected rail walk performance. During rail walk tests, performance also generally increased in Trial 2 compared with Trial 1, but not to a significant level (*P*>0.05). In a study performed after return from spaceflight, functional obstacle course time does improve over the course of six trials.^4^ However, these trials were all performed on the same test (albeit in clockwise and counterclockwise manner), and did not recover to pre-flight values until 7 days after landing. So while learning may have occurred in our study, it was likely insufficient to alter pre- to post-bed rest comparisons. Also, it is unclear how learning carries over between multiple different tests.

Another limitation is that other balance tests might be more sensitive to spaceflight and simulated spaceflight deconditioning. For example, balance impairment is more pronounced during diagnostic assessments that require active head movements.^9^ The added challenge of head tilting to stimulate vestibular function may help further discriminate balance abilities between the groups. A final limitation of this study is that rail balance testing may not be representative of astronauts’ needs for operational tasks or emergency egress on return to a gravitational environment.

In conclusion, treadmill exercise within LBNP attenuates losses in single-leg standing rail balance abilities in men during 30 days of simulated microgravity. However, this countermeasure did not have a significant effect on single-leg standing rail balance abilities in women, or on rail walking abilities in men or women. Similar exercise countermeasures within LBNP may help maintain standing balance control during spaceflight; yet this protective effect may, to some extent, depend on gender.

## Materials and methods

### Overall protocol

Eight pairs of healthy male identical twins (27±5 years, 173.6±11.5 cm, 67.2±10.0 kg) (mean±s.d.), and seven pairs of healthy female identical twins (24±3 years, 164.7±9.0 cm, 55.4±9.1 kg) volunteered to participate in this study. Identical twins were used to control for genetic responsiveness to bed rest and to increase statistical power.^44^ The performance of the countermeasure protocol was the only factor differentiating the two twins in each pair after bed rest. DNA samples were obtained using a cheek-swab kit, and used to confirm monozygosity by DNA polymorphism analysis for STR markers, D3 S1358, vWA, D16 S539, D2 S1338, D8 S1179, D21S11, D18 S51, D19 S433, Thol, and FGA. Before the study started, the Institutional Review Board at the University of California San Diego (UCSD) and the Committee for the Protection of Human Subjects at the NASA Johnson Space Center reviewed and approved all protocols prior to the start of the study. Before they participated in the study, subjects received verbal and written explanation of all procedures before providing informed written consent.

Subjects were admitted to the General Clinical Research Center (GCRC) at UCSD, where they received complete physical examinations from qualified physicians to confirm healthy status. Subjects completed a 6-day period of pre-bed rest ambulatory control during which they participated in familiarization and pre-bed rest testing protocols. Subjects then completed 30 days of strict 6° HDT bed rest during which they were not allowed to assume upright posture at any time, including during eating, drinking, urination, bowel movements, and transfer to and from testing and countermeasure sessions. However, subjects were supine (0°) during daily showers, body weight measures, and countermeasure sessions. In addition, subjects were allowed to lift their head with one elbow while eating. Subjects were allowed the use of one head pillow, but at no time were subjects allowed to raise their head above the level of their feet.

Subjects consumed a diet consisting of 55% carbohydrate, 30% protein, and 15% fat that was prepared by the Dietary Department staff of the UCSD GCRC. Diet records were maintained to ensure consistency throughout the pre-, during-, and post-bed rest periods. Each subject was prescribed an initial caloric consumption based on the Harris–Benedict equation, adjusting for self-reported activity level before admission to the GCRC admission (correction factor of 1.4–1.5). Body mass was measured each morning before breakfast at 0700 hours using a bedside scale (Century CC894; Hill-Rom, Batesville, IN, USA) and used to adjust caloric consumption such that subjects maintained their body mass within ±1.0 kg. In addition, sodium consumption was maintained at 3,500 mg per day, calcium consumption was targeted at 800–1,200 mg per day, and dietary fiber was prescribed at 25 g per day. Fluid intake was ad libitum, but no caffeinated or alcoholic beverages were allowed. Fluid and solid intakes and outputs were measured daily.

Subjects completed two pre-bed rest balance test sessions. The first test session served as familiarization, and only data from the second pre-bed rest test session were used as the baseline measure. The familiarization session was used to minimize learning effects during actual balance testing. The pre-bed rest familiarization and baseline test sessions were separated by at least 72 h. A third test session was completed immediately after 30 days of HDT bed rest. Balance testing consisted of single-leg rail balance tests and rail walk tests. Immediately before balance testing, subjects completed an orthostatic tolerance test to presyncope and a sprint test. Immediately after balance testing, subjects completed a graded exercise test to volitional fatigue. The day after balance testing (both before and after bed rest), knee flexion and extension strength and endurance were measured using an isokinetic dynamometer. All tests were performed in the same order and at the same time of day during familiarization, pre-bed rest, and post-bed rest periods. All tests were initiated at least 2 h after eating. Orthostatic tolerance, sprint speed, and exercise capacity data were reported elsewhere.^17–19^

### Exercise countermeasure

After pre-bed rest testing, one twin was randomly assigned to an exercise group that performed the LBNP countermeasure, while their sibling was assigned to a non-exercise control group. Exercise subjects performed 40 min of supine treadmill exercise within LBNP 6 days per week using target exercise intensities based on a percentage of their VO_2_ max, described in detail by Lee *et al.*^17^ These were the same protocols that successfully preserved upright exercise capacity in men during 15 days of HDT bed rest.^45^ Furthermore, because productive working time during spaceflight is at a premium, 40 min per day has been the recommended time for efficient exercise prescription in microgravity.^46^ Immediately after the exercise period, static LBNP was maintained at ~55 mm Hg for 5 min, while subjects rested supine with their legs extended against the treadmill belt and feet resisting the suction force of LBNP. This short LBNP period was used to simulate standing upright posture on Earth and to improve post-bed rest orthostatic tolerance, with the main rationale being that orthostatic stresses stimulate cardiovascular control mechanisms more strongly after exercise than without prior exercise.^19^ Exercise subjects were given 1 day of rest (no LBNP countermeasure) per week to allow recovery and prevent over-training.

The LBNP device consisted of a sealed partial-vacuum chamber in which subjects ran on a vertically oriented treadmill (PaceMaster SX-Pro, Aerobics, Little Falls, NJ, USA). This orientation allowed the negative pressure within the chamber to be the sole determinant for ground reaction forces experienced on the treadmill. Subjects were inserted horizontally into the LBNP chamber up to the level of the iliac crest, and a neoprene waist seal was used to enclose the lower-body in the chamber. Shoulder straps were attached to load the spine and prevent the seal from sliding down the subject’s body. A high-capacity vacuum created about 50–60 mm Hg of negative pressure to produce footward forces of 1.0–1.2 times body weight.^17^ If a subject showed signs or symptoms of presyncope during the countermeasure, they were encouraged to perform mild plantar flexion to activate the leg muscle pump and improve venous return to the heart. In the rare cases where presyncopal symptoms persisted after these measures, the static feet-supported LBNP was stopped. Once all symptoms abated, LBNP was restarted and continued at the prescribed levels to the completion of the countermeasure session.^19^

### Balance tests

Before and immediately after bed rest, subjects completed single-leg rail balance tests on a square wooden beam that was 7.6-cm wide and 4.9-m long. Tests were conducted in the same order before and after bed rest: left leg with eyes open, right leg with eyes open, left leg with eyes closed, and right leg with eyes closed. All tests ended when the subject stepped off the rail, touched the ground with their opposite foot, or reached a 30-s maximum. Three separate observers timed each trial using standard handheld stopwatches, and the three timers were averaged. Subjects who did not reach the 30-s maximum in Trial 1 performed a second test, Trial 2. Trial 1 balance times were used for statistical analysis because they were most representative of the effects of HDT bed rest and treadmill exercise within LBNP.

After completing the single-leg rail balance tests, subjects performed two rail walk trials, first with eyes open and then with eyes closed, on the same beam that was used for single-leg rail balance tests. In preliminary testing, the number of heel-to-toe steps required for each subject to reach the end of the beam was determined by having the subject walk alongside the beam. This number was used to determine the maximum number of steps that each subject could complete for each trial. Subjects were instructed to walk from one end of the rail to the other in the fastest manner possible using heel-to-toe steps. All tests ended when the subject stepped off the rail, touched the ground with one of their feet, or reached the end of the beam. Rail walk distance was scored by calculating the ratio of the number of steps taken in a trial over the number needed to reach the end of the beam, and multiplying this ratio by the length of the beam. Trial times were determined by averaging the times recorded by three measurers using standard handheld stopwatches. Trial 1 data were used for statistical analysis because they were most representative of the effects of HDT bed rest and treadmill exercise within LBNP.

Statistical significance was set at *P*<0.05. Shapiro–Wilk Normality test determined that our data were not normally distributed (*P*<0.05). Therefore, data were analyzed using nonparametric Wilcoxon and Mann–Whitney tests (IBM SPSS Statistics Version 21). The Wilcoxon test was used for related samples, which included between-group comparisons because the subjects were identical twins, and the Mann–Whitney test was used for unrelated samples. Single group data are presented as means±s.d., median, and 95% confidence intervals. Pre- to post-bed rest changes (Δ) in balance abilities were calculated by subtracting each subject’s post-bed rest balance data from their pre-bed rest balance data, and averaging these changes among their group. This was done for both the control and exercise groups (ΔControl group and ΔExercise group, respectively). Pre- versus post-bed rest comparisons are presented as mean changes (pre–post)±s.d.; 95% confidence intervals. Confidence intervals were generated using bootstrap distribution with at least 10,000 samples.

## Figures and Tables

**Table 1:** Single-leg rail balance time, rail walk time and rail walk distance statistics presented for each group before (Pre) and after (Post) bed rest, as well as the changes from pre- to post-bed rest

	*Single-leg rail balance time (s)*			*Rail walk time (s)*			*Rail walk distance (m)*		
	*Mean*±*s.d.*	*Median*	*95% CI*	*Mean*±*s.d.*	*Median*	*95% CI*	*Mean*±*s.d.*	*Median*	*95% CI*
*Males (*n*=8)*									
Pre- bed rest control	22.8±11.5	30.0	18.7–26.5	8.3±5.2	7.3	6.1–11.0	3.0±1.8	2.9	2.2–3.9
Pre- bed rest exercise	20.2±12.1	30.0	16.1–24.2	9.0±5.7	7.3	6.4–11.8	3.3±1.8	4.2	2.4–4.1
Post-bed rest control	13.2±11.8	7.4	9.3–17.3	5.4±3.2	4.5	4.1–7.1	1.7±1.4	1.4	1.1–2.4
Post-bed rest exercise	16.6±12.8	12.4	12.3–20.9	5.7±3.7	4.3	4.1–7.6	2.1±1.5	1.6	1.4–2.8
Δ (pre–post) control	9.5±13.8	—	3.7–14.9	2.9±4.7	—	0.1–5.9	1.3±1.8	—	0.2–2.4
Δ (pre–post) exercise	3.5±12.0	—	2.4–9.4	3.2±6.4	—	0.1–6.5	1.2±1.6	—	0.1–2.3
*Females (*n*=7)*									
Pre- bed rest control	20.9±11.8	30.0	16.4–25.1	11.1±7.4	10.1	7.7–15.1	3.7±1.7	4.9	2.8–4.9
Pre- bed rest exercise	20.9±11.6	30.0	16.6–25.0	9.2±5.9	7.7	6.6–12.6	3.1±1.9	3.4	2.1–4.1
Post-bed rest control	12.6±11.8	5.9	8.4–17.1	7.7±7.1	6.6	4.8–11.8	2.5±1.8	1.8	1.7–3.4
Post-bed rest exercise	16.0±12.9	10.4	11.3–20.7	5.5±3.0	4.5	4.1–7.1	2.2±1.7	1.5	1.4–3.0
Δ (pre–post) control	8.3±12.6	—	2.1–14.3	3.5±6.5	—	−1.9–8.4	1.2±1.4	—	−0.1–2.4
Δ (pre–post) exercise	4.9±8.8	—	1.5–11.2	3.8±6.2	—	0.7–7.3	0.9±1.5	—	−0.4–2.2

Abbreviation: CI, confidence interval.
